# Amentoflavone reverses epithelial‐mesenchymal transition in hepatocellular carcinoma cells by targeting p53 signalling pathway axis

**DOI:** 10.1111/jcmm.18442

**Published:** 2024-06-06

**Authors:** Hui‐Ying Jian, Jing‐Ting Zhang, Zhuo Liu, Zhen Zhang, Pu‐Hua Zeng

**Affiliations:** ^1^ Hunan University of Chinese Medicine Hunan China; ^2^ Hunan Provincial Hospital of Integrated Traditional Chinese and Western, Cancer Research Institute of Hunan Academy of Traditional Chinese Medicine Hunan Academy of Chinese Medicine Hunan China

**Keywords:** Amentoflavone, bioinformatics, epithelial‐mesenchymal transition, hepatocellular carcinoma, network pharmacology, p53 signalling pathway axis

## Abstract

Epithelial‐mesenchymal transition (EMT) and its reversal process are important potential mechanisms in the development of HCC. *Selaginella doederleinii* Hieron is widely used in Traditional Chinese Medicine for the treatment of various tumours and Amentoflavone is its main active ingredient. This study investigates the mechanism of action of Amentoflavone on EMT in hepatocellular carcinoma from the perspective of bioinformatics and network pharmacology. Bioinformatics was used to screen Amentoflavone‐regulated EMT genes that are closely related to the prognosis of HCC, and a molecular prediction model was established to assess the prognosis of HCC. The network pharmacology was used to predict the pathway axis regulated by Amentoflavone. Molecular docking of Amentoflavone with corresponding targets was performed. Detection and evaluation of the effects of Amentoflavone on cell proliferation, migration, invasion and apoptosis by CCK‐8 kit, wound healing assay, Transwell assay and annexin V‐FITC/propidium iodide staining. Eventually three core genes were screened, inculding NR1I2, CDK1 and CHEK1. A total of 590 GO enrichment entries were obtained, and five enrichment results were obtained by KEGG pathway analysis. Genes were mainly enriched in the p53 signalling pathway. The outcomes derived from both the wound healing assay and Transwell assay demonstrated significant inhibition of migration and invasion in HCC cells upon exposure to different concentrations of Amentoflavone. The results of Annexin V‐FITC/PI staining assay showed that different concentrations of Amentoflavone induces apoptosis in HCC cells. This study revealed that the mechanism of Amentoflavone reverses EMT in hepatocellular carcinoma, possibly by inhibiting the expression of core genes and blocking the p53 signalling pathway axis to inhibit the migration and invasion of HCC cells.

## 1 INTRODUCTION

Primary liver cancer (PLC) represents a formidable malignancy characterized by substantial histological and biological diversity. According to GLOBOCAN 2020 data, there were 910 thousand new cases of liver cancer and 830 thousand associated deaths worldwide.^1^ PLC encompasses malignant tumours originating from hepatocytes or intrahepatic bile duct epithelial cells, manifesting as three distinct pathological types: hepatocellular carcinoma (HCC), intrahepatic cholangiocarcinoma (ICC) and combined HCC‐ICC (cHCC‐ICC). Notably, HCC constitutes approximately 90% of cases within this category. The global landscape continues to grapple with significant challenges posed by HCC, evident in its prevalence and mortality rates. The estimated 5‐year survival rate for HCC stands at a mere 18%, underscoring the gravity of the situation.^2^ PLC stands as a prevalent malignant tumour in China, where it constitutes 45.3% of new cases and 47.1% of related deaths, contributing to approximately half of the global incidence (389 thousand, ranking fourth) and deaths (336 thousand, ranking second).^3^ Presently, the median survival for liver cancer in China is approximately 23 months,^4^ while the median survival for advanced liver cancer is a mere 6 months.^1^, ^5^ This places an exceptionally burdensome load on both patients and society, emphasizing the profound challenges associated with the disease. The treatment modalities for PLC vary depending on the disease stage and may include resection, radiofrequency ablation, interventional embolization, radiotherapy, systemic chemotherapy, as well as molecular targeted therapy and immunotherapy. However, due to the insidious onset, high malignancy, and rapid progression of PLC, a considerable number of patients are diagnosed at the intermediate to advanced stages, precluding the opportunity for surgical intervention. Despite the approval and clinical testing of numerous drugs for advanced hepatocellular carcinoma (HCC), both the median progression‐free survival (PFS) and overall survival (OS) rates remain disheartening.^6^ The diagnostic and therapeutic landscape for PLC is notably challenging, given the severity of the disease.

The main reason for the poor prognosis of HCC is that HCC is prone to metastasis. There are many mechanisms associated with tumorigenesis and metastasis, and EMT and its reversal process are the most important potential mechanisms.^7^ Emerging evidence suggests that epithelial‐mesenchymal transition (EMT) is activated during the development, growth, progression, and metastasis of hepatocellular carcinomas.^8^ Therefore, intervening in this process may be a novel strategy to prevent liver cancer metastasis. TGF‐β signalling pathway plays an important role in the development of hepatocellular carcinoma. TGF‐β may induce a partial EMT in some epithelial HCC cells, increasing the expression of mesenchymal genes but maintaining epithelial gene expression.^9^ A mesenchymal, migratory, and invasive phenotype in HCC cells correlates with high autocrine TGF‐β expression.^10^ Bioinformatics is playing an increasingly important role in almost all aspects of drug discovery, evaluation, and development. Molecular docking can reveal the molecular mechanism of drug treatment of diseases, elevate pharmacological effects to the molecular level, and lay the material foundation for the study of drug action mechanisms.^11^

A large number of phytochemicals are thought to modulate several molecular and metabolic processes, such as cell cycle regulation, apoptosis activation, angiogenesis, and metastasis inhibition, all of which can halt cancer progression.^12^, ^13^, ^14^ Amentoflavone [8‐[5‐(5,7‐dihydroxy‐4‐oxochromen‐2‐yl)‐2‐hydroxyphenyl]‐5,7‐dihydroxy‐2‐(4‐hydroxyphenyl) chromene‐4‐one, AF], a natural biflavonoids isolated from the medicinal plant *Selaginella doederleinii* Hieron,^15^ is also its main active ingredient. Amentoflavone is involved in anti‐cancer activity by mediating various signalling pathways such as extracellular signal‐regulated kinase (ERK), nuclear factor kappa‐B (NF‐κB), and phosphoinositide 3‐kinase/protein kinase B (PI3K/Akt).^16^, ^17^, ^18^, ^19^, ^20^ However, no studies exist regarding the effects of Amentoflavone on epithelial‐mesenchymal transition cells in HCC.

**FIGURE 1:**
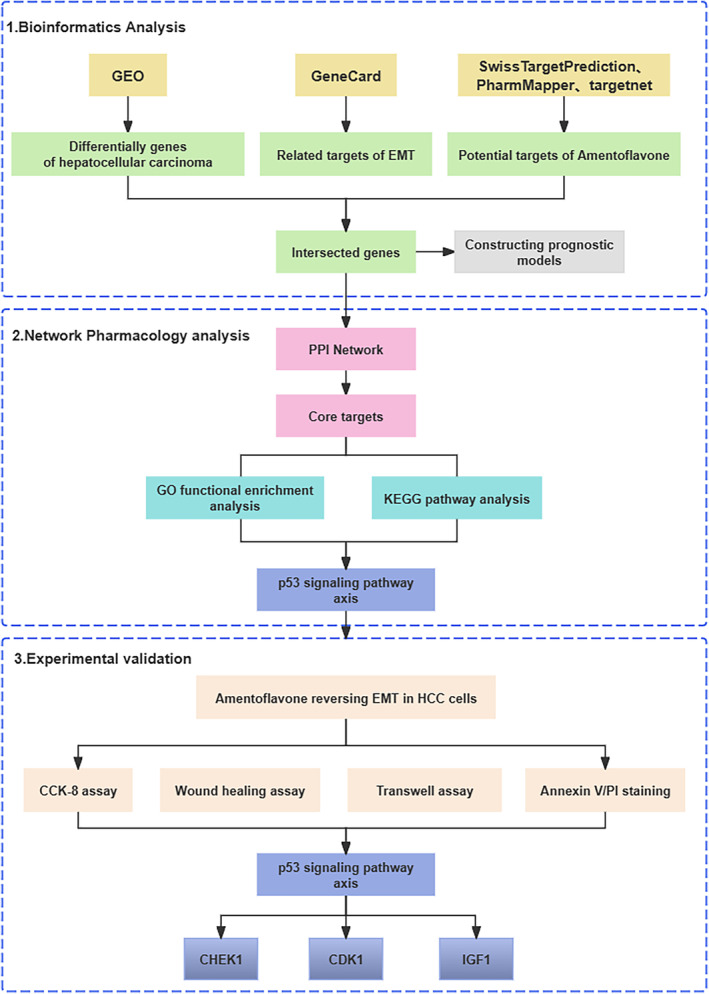
The schematic diagram of the research methodology and workflow of the present study of Amentoflavone acting on EMT in hepatocellular carcinoma.

In this study, we investigated EMT‐related genes and applied liver cancer data to The Cancer Genome Atlas (TCGA) for bioinformatics analysis. Then Amentoflavone regulated EMT genes, which are closely related to the prognosis of hepatocellular carcinoma, were screened and molecular prediction models were established to assess the prognosis of hepatocellular carcinoma. For the screened core genes then network pharmacology and molecular docking techniques were applied to investigate the targets and pathways of action of Amentoflavone to reverse EMT in hepatocellular carcinoma. And was validated in in vitro experiments (Figure 1).

## 2 METHODS

### 2.1 Bioinformatics analysis of Amentoflavone on hepatocellular carcinoma through modulation of EMT

#### 2.1.1 Collection of differential genes of hepatocellular carcinoma

The keyword ‘Hepatocellular carcinoma’ was searched in the GEO database (https://www.ncbi.nlm.nih.gov/geo/). The GSE84402 dataset was retrieved, and 14 transcriptome data from hepatocellular carcinoma tissues and their paraneoplastic normal tissues were downloaded and subjected to variance analysis. GSE84402 was analysed for differences using the R language limma package. *p* < 0.05 and |log2(FC)| >2 were considered statistically significant.

#### 2.1.2 Collection of genes modulating the epithelial‐mesenchymal transition

The keywords ‘Epithelial‐mesenchymal transition’ and ‘EMT’ were searched in the GeneCards database (https://www.genecards.org/), and duplicate values as targets for modulating the EMT were removed.

#### 2.1.3 Targets for drug treatment of diseases

The keywords ‘Amentoflavone’ was searched in the Pubmed database. The compound was subjected to target prediction in the SwissTarget Prediction (http://www.swisstargetprediction.ch), PharmMapper (http://www.lilab‐ecust.cn/pharmmapper/) and targetnet (http://targetnet.scbdd.com). Targets related to Amentoflavone were acquired. After duplicates were removed, all targets were converted to gene names using the Uniprot database (https://www.uniprot.org/).

#### 2.1.4 Acquisition of intersecting genes

The intersection of differential genes in the GSE84402 dataset, EMT‐regulated genes, and related targets of Amentoflavone were obtained using the ggplot2 package for the R software (4.2.1). These intersecting genes were identified as key genes in Amentoflavone regulation of EMT and thus hepatocellular carcinoma development.

#### 2.1.5 Validation of differential expression of intersecting genes in patients with hepatocellular carcinoma

RNAseq data of the STAR process of the TCGA‐LIHC (hepatocellular carcinoma) project were downloaded from the TCGA database (https://portal.gdc.cancer.gov) and extracted in FPKM format to compare the gene expression differences between hepatocellular carcinoma tissues and paraneoplastic liver tissues. Statistical analysis was performed using R software. If the gene expression data in the TCGA dataset conformed to normality, paired/unpaired samples *t*‐tests were used, if the gene expression data in the TCGA dataset did not conform to normality, paired/unpaired samples rank sum tests were used. *p* < 0.05 was considered statistically significant.

#### 2.1.6 Prognostic analysis of intersecting genes in patients with hepatocellular carcinoma

RNAseq data of the STAR process of the TCGA‐LIHC (hepatocellular carcinoma) project were downloaded from the TCGA database (https://portal.gdc.cancer.gov) and extracted in FPKM format. After removing samples with no clinical information, the 14 previously screened genes were screened for higher prognostic correlation with hepatocellular carcinoma using lasso regression and cross‐testing. The cleaned data were analysed to obtain lambda values, and partial likelihood deviance and visualized using the ‘glmnet’ package of R software.

#### 2.1.7 Construction and validation of the core gene risk scoring system

The three core genes (NR1I2, CDK1, CHEK1) screened in the previous step were subjected to multifactorial Cox regression using the R software, and risk scores were calculated using survival outcome and survival time as dependent variables. Risk score calculation formula:

$$\text{Riskscore}=\sum_{i=1}^{n}\text{coefficients}\times \text{expressiongenes}\left(i\right).$$

Evaluation of 1‐, 3‐, and 5‐year survival prediction efficacy for risk score using the timeROC Package of R software. According to the cut‐off value of the core gene risk score time‐ROC, patients with hepatocellular carcinoma in the TCGA‐LIHC cohort were divided into high‐ and low‐risk groups, and Kaplan–Meier curves were plotted to compare the survival prognosis of the high‐ and low‐risk groups using log‐rank statistical analysis.

#### 2.1.8 Construction and validation of nomogram of core genes

One‐way and multifactorial Cox regression analyses were performed using the R software, and *p* < 0.1 for one‐way Cox regression was used as the condition for inclusion in multifactorial Cox regression, OS was the dependent variable, and gender, age, Pathologic staging, and risk score of hepatocellular carcinoma patients in the TCGA database were included as the independent variables and HR, 95% CI and *p*‐value were calculated to evaluate the gender and age, Pathologic staging, and risk score as independent prognostic factors for survival in hepatocellular carcinoma. The nomogram prognostic model was constructed using the ‘rms’ and ‘survival’ packages of R software. The nomogram prognostic model was constructed to predict the survival of hepatocellular carcinoma patients at 1, 3, and 5 years by combining the risk score of hepatocellular carcinoma patients and their clinical information. Calibration analysis was performed to validate the nomogram prognostic model using the ‘rms’ package and ‘survival’ package of the R software to assess the predictive ability of the nomogram prognostic model.

### 2.2 Network pharmacology analysis of Amentoflavone on hepatocellular carcinoma through modulation of EMT

#### 2.2.1 Construction of the PPI network

To clarify the interaction between the intersection genes, the 14 regulated genes from the previous screening were imported into the STRING online platform (https://cn.string‐db.org/), the protein type was set as ‘Homo sapiens’, the confidence was set >0.15, and the other parameters remained the default, to obtain the protein interaction relationship.

#### 2.2.2 GO functional enrichment analysis and KEGG pathway analysis

The gene information of the 14 regulated genes was used for GO and KEGG enrichment analysis using the ‘ClusterProfiler’ and ‘org.Hs.eg.db’ packages of R software. *p* < 0.05 was used as the screening index.

#### 2.2.3 Molecular docking verification of Amentoflavone with target proteins

The 3D structure of Amentoflavone was downloaded from the PubChem database, and ChemBio 3D software was used for energy optimization to obtain the 3D structure of the compound, which was stored in mol2 format. The 3D crystal structure of the target protein with resolution below 2.5 Å was downloaded from the PDB (https://www.rcsb.org/) database and saved it in PDB format. Water molecules and small molecular ligands were removed in AudockTools software. The MGLTools support tool of Audock vina software is used to hydrogenate the corresponding, complete the selection of receptors, and set the docking active centre (including the entire protein molecule) through the grid box function in the software. Finally, the docking was completed by the vina molecular docking function. The smaller the molecular binding energy (affinity) is, the better the binding activity between the protein and compound.

### 2.3 Cell lines and culture

Human liver cancer MHCC97H cells were gifted from the Hunan Key Laboratory of TCM Prescription and Syndromes Translational Medicine, Human liver cancer MHCCLM3 cells were purchased from Guangzhou Cellcook Biotech Co., Ltd and were used at the 5th passage for all experimental procedures. The cells were incubated at 37°C in a 5% CO_2_ water jacket incubator (Thermo Fisher Scientific) using DMEM with supplements of 10% FBS and 1% Penicillin–Streptomycin Solution.

#### 2.3.1 Chemicals and reagents

Amentoflavone (AF, purity ≥98%, B21229) was purchased from Shanghaiyuanye BioTechnology Co., Ltd. TGF‐β1 (CA59) was purchased from Novoprotein. Dulbecco's modified eagle medium (DMEM, PM15210), phosphate‐buffered saline (PBS, PB180327), fetal bovine serum (FBS, 164210), Penicillin–Streptomycin Solution (PB180120) were purchased from Procell Life Science&Technology Co., Ltd. (Wuhan, China). 0.25% Trypsin was purchased from ECTOP. Matrigel (356234) was purchased from Corning (New York, USA). Annexin V‐FITC/PI Apoptosis Kit (K2003) was purchased from APExBIO (Houston, USA). RIPA Lysis Buffer (Strong) (CW2333) was purchased from CWBIO (Beijing, China). BCA protein concentration measurement Kit (P0010) was purchased from Beyotime Biotechnology Co., Ltd. (Shanghai, China).

E‐cadherin polyclonal antibody (20874‐1‐AP), N‐cadherin polyclonal antibody (22018‐1‐AP), Vimentin polyclonal antibody (10366‐1‐AP), CDK1 polyclonal antibody (10762‐1‐AP), CHEK1 polyclonal antibody (25887‐1‐AP), and IGF1 polyclonal antibody (28530‐1‐AP) were purchased from Proteintech (Wuhan, China). Beta Actin Antibody (AF7018) was purchased from Affinity Biosciences (Jiangsu, China).

#### 2.3.2 Cytotoxicity assay

Cytotoxicity of Amentoflavone on HCC cells was measured by CCK‐8 assay. Logarithmically growing MHCC97H cells and MHCCLM3 cells were seeded at 5 × 10^3^ per well in 96‐well plates. Once cells adhered properly, they were treated with various concentrations of Amentoflavone (0, 100, 200, 300, 400, and 500 μmol/L) and incubated for 24 h at 37°C with 5% CO_2_. Subsequently, the medium was removed, and 10 μL of CCK‐8 solution was added to each well, followed by further incubation at 37°C for 1 h in the culture chamber. Cell viability was finally determined by measuring absorbance at 450 nm. IC_50_ is a measure of the drug's potency in inhibiting a specific biological activity or cell growth. The IC_50_ was determined using GraphPad Prism 9.0.

#### 2.3.3 Establishment of HCC EMT cell model

The HCC EMT model cells were established with 20 ng/mL TGF‐β1 intervention at 48 h. Logarithmic growth phase MHCC97H and MHCCLM3 EMT model cells were used in the experiments.

#### 2.3.4 Groups and treatment

EMT model cells in the logarithmic growth phase were divided into four groups: the EMT model group, AF‐L group, AF‐M group, and AF‐H group, wherein the culture was added to the EMT model group and cultured for 24 h. The AF groups were exposed to various concentrations of Amentoflavone (100, 200, or 300 μmol/L) for 24 h. The above experimental groups were compared with the control group for EMT of HCC. The Control group was added to the culture and cultured for 24 h.

#### 2.3.5 Wound healing assay

MHCC97H and MHCCLM3 cells were plated at a density of 2 × 10^4^ cells/well in 6‐well plates until the formation of a confluent monolayer. After 24 h of culturing, using a 200 μL pipette, a clean scratch wound was created across the centre of each well. Subsequently, the floating cells were washed away by PBS, and fresh DMEM with supplements of 10% FBS and various drugs were added to each well. The 6‐well plates were then incubated in a CO_2_ incubator, allowing for the migration of cells. Each wound was photographed at predetermined time intervals (24 h and 48 h), and the migration was determined using Image J software. Migration (%) = (initial scratch width−final scratch width)/initial scratch width × 100%.

#### 2.3.6 Transwell assay

MHCC97H and MHCCLM3 cells were suspended in serum‐free DMEM medium and seeded into the upper chambers at a concentration of 1 × 10^4^ cells in 100 μL of cell suspension. The lower chambers were filled with medium supplemented with 10% FBS, serving as a chemoattractant to facilitate cell invasion. Subsequently, the upper chambers were treated with various drugs and incubated for 48 h in a humidified atmosphere at 37°C with 5% CO_2_. The Matrigel and non‐invading cells on the upper surface of the membrane were gently removed using a cotton swab, while the invading cells were fixed, stained, and visualized under a microscope. The number of invading cells in each group was quantified using Image J software.

#### 2.3.7 Cellular apoptosis assay

The apoptotic cells in each group were estimated using the apoptosis detection kit according to the manufacturer's instructions. MHCC97H and MHCCLM3 cells were digested with trypsin without EDTA, centrifuged at 300 g for 5 min, and the cells were collected. Cells were washed twice with pre‐cooled PBS, centrifuged at 300 g for 5 min, and resuspended in 500 μL 1× binding buffer (1–5 × 10^5^ cells/group). Subsequently, 5 μL Annexin V‐FITC and 5 μL propidium iodide (PI) staining solution were added to the cell suspension, gently mixed, and incubated in the dark at room temperature for 20 min. Flow cytometry analysis of stained cells was performed within 1 h. The formula for calculating the cell apoptosis rate is: (number of apoptotic cells/total number of observed cells) × 100%.

#### 2.3.8 Western blotting

MHCC97H and MHCCLM3 specimens were collected, and total protein was extracted utilizing RIPA Lysis Buffer containing 1% PMSF. After mixing, the cell sample was shaken at 4°C for 30 min and then centrifuged at 14,000 g and 4°C for 10 min. The protein concentration of the supernatant was measured by the BCA method, and adjust the protein concentration of each sample to 1.5 mg/mL with 5 × SDS Loading buffer. The samples were subjected to 100°C heating for 10 min for denaturation, and then let them cool for later use. Add a certain volume of each protein sample, resulting in a 30 mg protein load per sample. After SDS‐PAGE electrophoresis, the proteins were transferred from SDS polyacrylamide gels to nitrocellulose (PVDF) membranes. These membranes were blocked with 5% nonfat milk at room temperature for 1 h before being incubated overnight at 4°C with primary antibodies [anti‐E‐cadherin (1: 5000), anti‐N‐cadherin (1:1000), anti‐Vimentin (1:3000), anti‐p53 (1:2000), anti‐CHEK1 (1:1000), anti‐CDK1 (1:2000), anti‐IGF1 (1:3000), and anti‐β‐actin (1:2000)] and then incubated overnight at 4°C. The membranes were then washed three times with TBST (10 min each) before being incubated with HRP‐conjugated secondary antibodies (1:10000) for 60 min. Following this, the membranes were washed three times with TBST and visualized utilizing chemiluminescence with an ECL detection system. The relative protein expression levels were then quantified using Image J software.

### 2.4 Statistical analysis

The author will be notified during the typesetting of the final article if this is the case. Statistical analysis was performed using SPSS 22.0. The measures all satisfy a normal distribution. All data were expressed as mean ± standard error (SE) with a sample size of *n* = 3. To compare differences among multiple groups, a one‐way analysis of variance (anova) was employed. In cases where the data satisfied the assumptions of normality and homogeneity of variances, anova was conducted, and two‐by‐two comparisons between multiple groups were performed using the LSD‐*t*‐test. However, if the assumption of homogeneity of variances was not met, the Welch test was utilized. A *p* < 0.05 was statistically significant.

## 3 RESULTS

### 3.1 Screening of key genes, construction and evaluation of prognostic models

**FIGURE 2:**
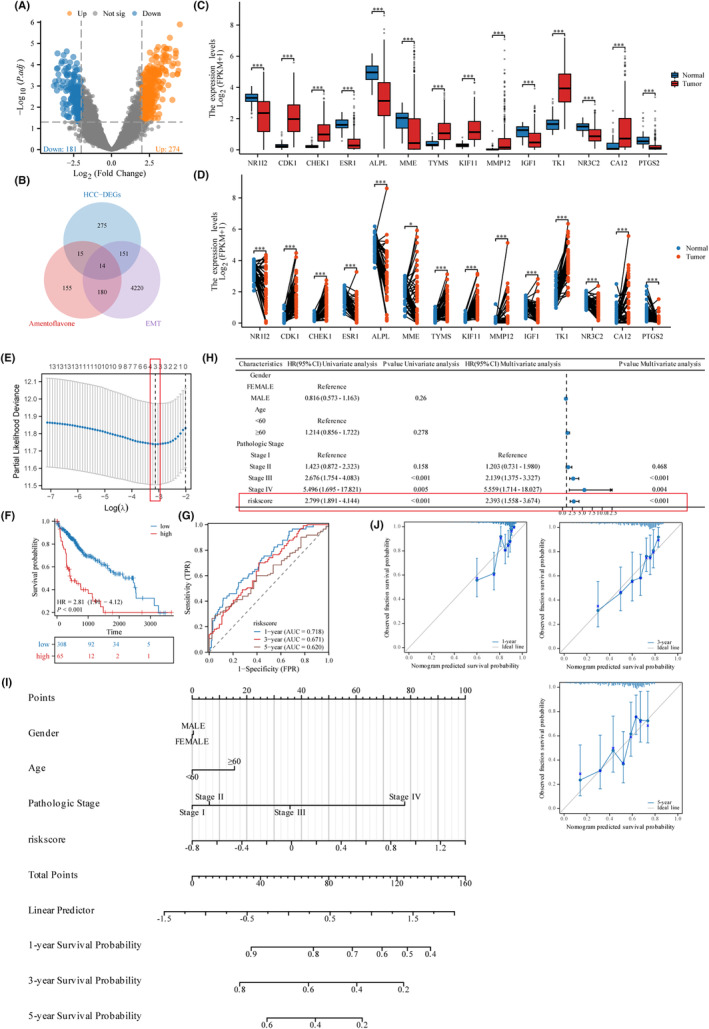
The screening of key genes, construction and evaluation of prognostic models. (A) The volcano map of hepatocellular carcinoma differential genes; (B) the Venn diagram of intersecting genes; (C) unpaired sample *t*‐test results for intersecting genes; (D) paired‐sample *t*‐test results for intersecting genes. (E) The visualization results of Lasso regression for intersecting genes; (F) time‐dependent receiver operating characteristic (ROC) curve of the Risk scoring system for core genes; (G) Kaplan–Meier survival of the Risk scoring system for core genes; (H) forest plots for single and multifactorial Cox regression analysis of core genes; (I) nomogram of the predictive model; (J) calibration analysis of nomogram prediction.

The results of the differential analysis indicated that 455 differential genes were obtained in the GSE84402 dataset for samples of hepatocellular carcinoma tissues compared with those of normal liver tissues, of which 181 genes were differentially down‐regulated and 274 genes were differentially up‐regulated in expression (Figure 2A). A total of 4574 related genes were obtained by searching the Gene Card database with the keyword ‘EMT’. The smiles number of ‘Amentoflavone’ and the SDF format were obtained from the PubChem database. Ten predicted targets were obtained from SwissTarget Prediction, 289 from PharmMapper, 104 from Target Net, and 364 genes after deleting duplicates. The above three parts of the genes were taken to be intersected and a total of 14 genes were obtained, namely NR1I2, CDK1, CHEK1, ESR1, ALPL, MME, TYMS, KIF11, MMP12, IGF1, TK1, NR3C2, CA12 and PTGS2 (Figure 2B). The next step was to validate the differential expression of intersecting genes in patients with hepatocellular carcinoma. In the TCGA‐LIHC cohort, hepatocellular carcinoma tissues were tested against paracancerous liver tissues in unpaired/paired samples. Among them, CDK1, CHEK1, TYMS, KIF11, MMP12, TK1 and CA12 were differentially highly expressed in hepatocellular carcinoma tissues. NR1I2, ESR1, ALPL, MME, IGF1, NR3C2 and PTGS2 were differentially low expressed in hepatocellular carcinoma tissues (Figure 2C,D).

Among the 14 intersecting genes, lasso regression was used to screen for genes with higher prognostic correlation with hepatocellular carcinoma, and a crossover test was performed to screen for three genes with higher prognostic correlation with patients—NR1I2, CDK1, and CHEK1. The cleaned data is analysed to obtain λ‐values, and partial likelihood of deviance, and the data is visualized. As shown in Figure 2E, The optimal lambda value (dashed line on the left) corresponds to a variable number of 3, validating that the three genes screened were highly associated in the prognostic model.

Next, using survival outcomes and survival time as dependent variables, single and multifactorial Cox regression analyses were performed on these three core genes (NR1I2, CDK1 and CHEK1), Coef correlation coefficients of the Cox risk model for the key genes were computed, and the expression values of NR1I2, CDK1, and CHEK1 in hepatocellular carcinoma patients in the TCGA‐LIHC cohort were included to compute the risk scores of the core genes, and to construct the risk‐scoring system for the combination of the three genes. That is, Risk Score = −0.114 × NR1I2 (exp) + 0.125 × CDK1 (exp) + 0.355 × CHEK1 (exp) − 0.414. ROC curve of the risk scoring system for core genes showed that AUC = 0.718, 0.671 and 0.620 at 1, 3 and 5 years, all of which were good predictors of patient survival (Figure 2F). Of these, 1 year had the best prognostic efficacy, so the time‐ROC curve cut‐off value for 1 year was chosen (cut‐off = 0.424). Risk scores were calculated for each hepatocellular carcinoma patient in the TCGA‐LIHC cohort, and the hepatocellular carcinoma patients were divided into high‐ and low‐risk groups according to the optimal cut‐off value of 1 year in the time‐ROC curves, with 308 cases in the low‐risk group and 65 cases in the high‐risk group. Comparison of the difference in survival between the high‐ and low‐risk groups showed that the high‐risk group had a worse survival prognosis than the low‐risk group, with a statistically significant difference of HR = 2.81 (Figure 2G, 95% CI = 1.91–4.12, *p* < 0.01).

Independent prognostic analysis of risk scores and clinical information in patients with hepatocellular carcinoma from the TCGA database. The results of univariate Cox analysis showed that the pathologic stage and risk score were risk factors for the prognosis of patients with hepatocellular carcinoma. Further multifactorial Cox analysis showed that only risk score was an independent risk factor for prognosis in patients with hepatocellular carcinoma [Figure 2 H, HR = 2.393, 95% CI = (1.558–3.674), *p* < 0.001]. Combining age, gender, pathologic stage, and risk score of hepatocellular carcinoma patients in the TCGA‐LIHC cohort to construct nomograms predicting 1‐, 3‐, and 5‐year survival of hepatocellular carcinoma patients. The C‐index for this nomogram is calculated to be 0.690 (0.664–0.715), which has good predictive power (Figure 2I). Calibration analysis showed that the scatter points were close to the ideal line, indicating that the 1‐, 3‐, and 5‐year survival probabilities predicted by the nomogram plot were in good agreement with the actual observations, and the model validity was good (Figure 2J).

### 3.2 PPI network construction, GO and KEGG enrichment analysis results

**FIGURE 3:**
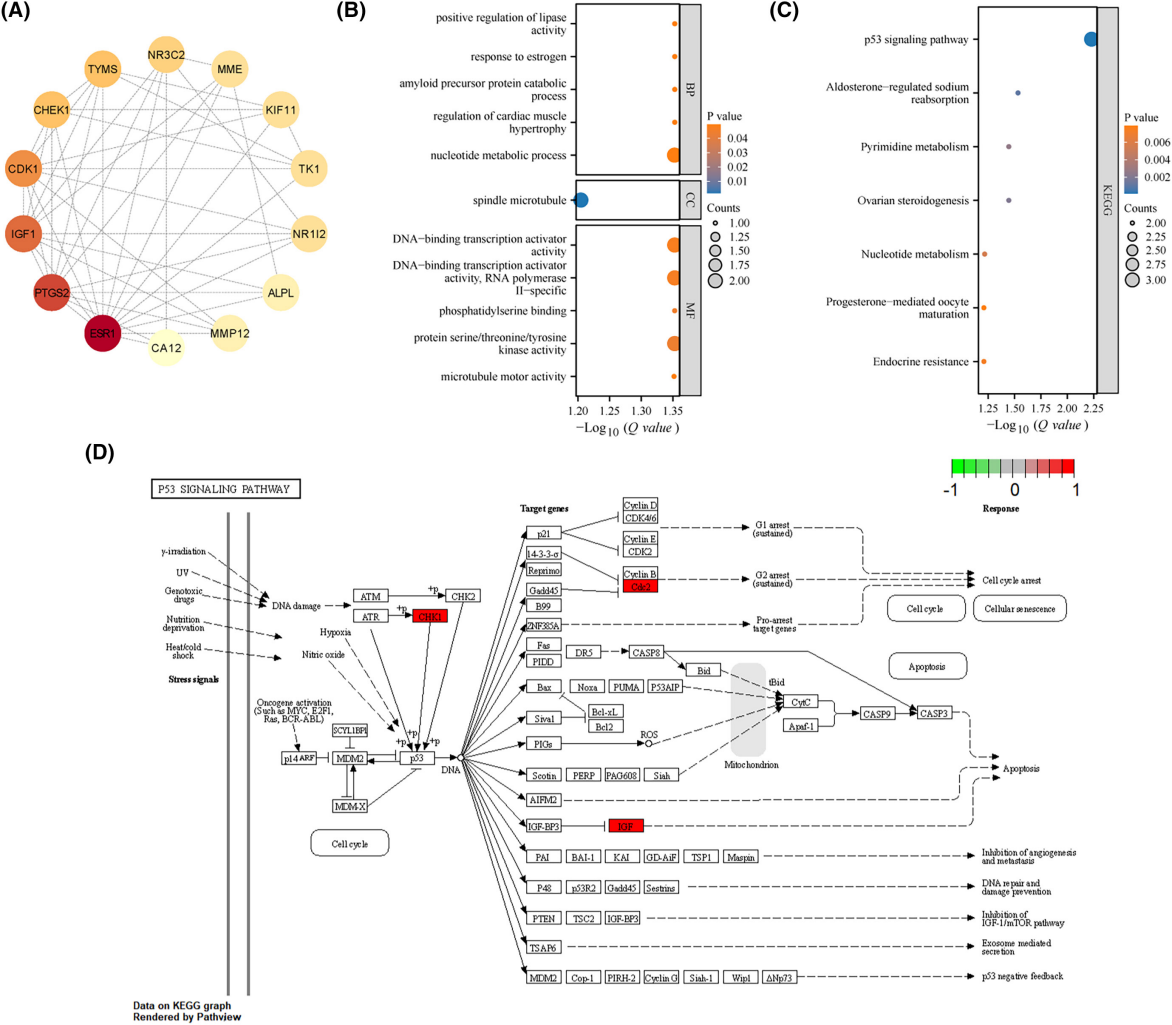
Network Pharmacology analysis results of Amentoflavone on Hepatocellular Carcinoma through modulation of EMT. (A) PPI network; (B) GO enrichment analysis (BP, biological process; CC, cell components; MF, molecular function); (C) KEGG enrichment analysis; (D) Gene enrichment map of the p53 signalling pathway (CHK1 also known as CHEK1, Cdc2 also known as CDK1, IGF is IGF1).

The 14 regulated gene information was imported into the STRING online platform, and the results were imported into Cytoscape 3.9.1 software. PPI results showed a total of 47 histone interactions between 14 targets (Figure 3A). Next, the gene information of the 14 regulated genes was used for GO and KEGG enrichment analysis using the ‘ClusterProfiler’ and ‘org.Hs.eg.db’ packages of R software. *p* < 0.05 was used as the screening index, and the top five of BP, MF, CC and KEGG were selected for visualization according to the *Q*‐value. A total of 590 GO enrichment entries were obtained, including biological processes (BP, 573), cell components (CC, 1), and molecular functions (MF, 16). The main enriched biological processes included nucleotide metabolic process， regulation of cardiac muscle hypertrophy, amyloid precursor protein catabolic process, response to oestrogen and positive regulation of lipase activity; the main enriched cell component was spindle microtubule. The main enriched molecular functions included microtubule motor activity, protein serine/threonine/tyrosine kinase activity, phosphatidylserine binding, DNA‐binding transcription activator activity, RNA polymerase II‐specific, and DNA‐binding transcription activator activity. The results of KEGG pathway enrichment analysis showed that genes were mainly enriched in the p53 signalling pathway, aldosterone‐regulated sodium reabsorption, ovarian steroidogenesis and pyrimidine metabolism. The five pathways with the smallest *p*‐values of biological processes, cell components and molecular functions in GO enrichment analysis and KEGG enrichment (Figure 3B,C). Finally, the p53 signalling pathway axis was visualized and enriched to three core genes, CHEK1, CDK1 and IGF1 (Figure 3D).

### 3.3 Molecular docking results

**FIGURE 4:**
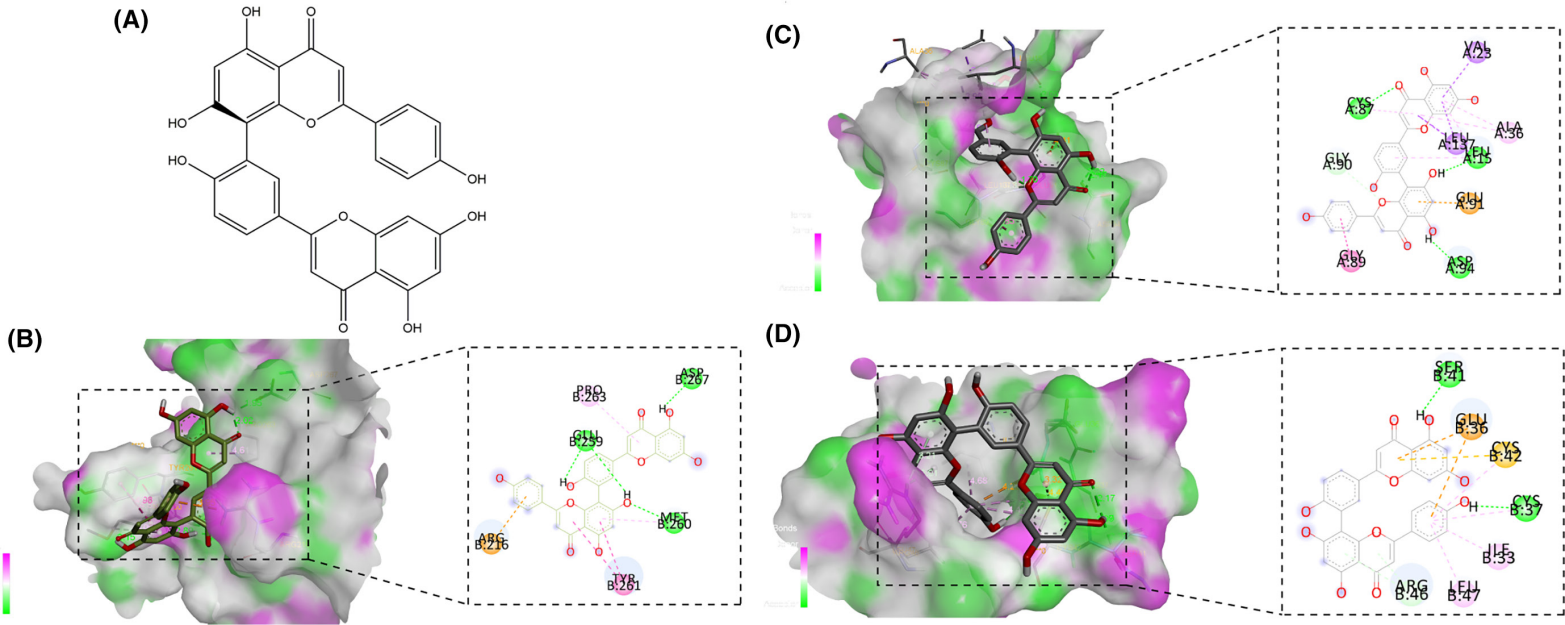
Docking diagram of Amentoflavone with core targets. (A) 2D chemical structure of Aamentoflavone; (B) Docking diagram of Amentoflavone and CHEK1; (C) Docking diagram of Amentoflavone and CDK1; (D) Docking diagram of Amentoflavone and IGF1.

AutoDock Vina software was used for molecular docking of the core gene target protein (CDK1, CHEK1 and IGF1) and the interaction of Amentoflavone. It is generally believed that the lower the binding energy of the ligand and receptor, the better the binding force. When the binding energy is less than 0 kcal/mol, the two can spontaneously combine. When two and more hydrogen bonds (green orbs) appear in the 2D plot of the molecular docking visualization, the two can spontaneously combine. According to the molecular docking results, Amentoflavone has good molecular docking efficacy with core genes. Visualization of molecular docking is shown in Figure 4.

### 3.4 Establishment and verification of HCC EMT cell model

**FIGURE 5:**
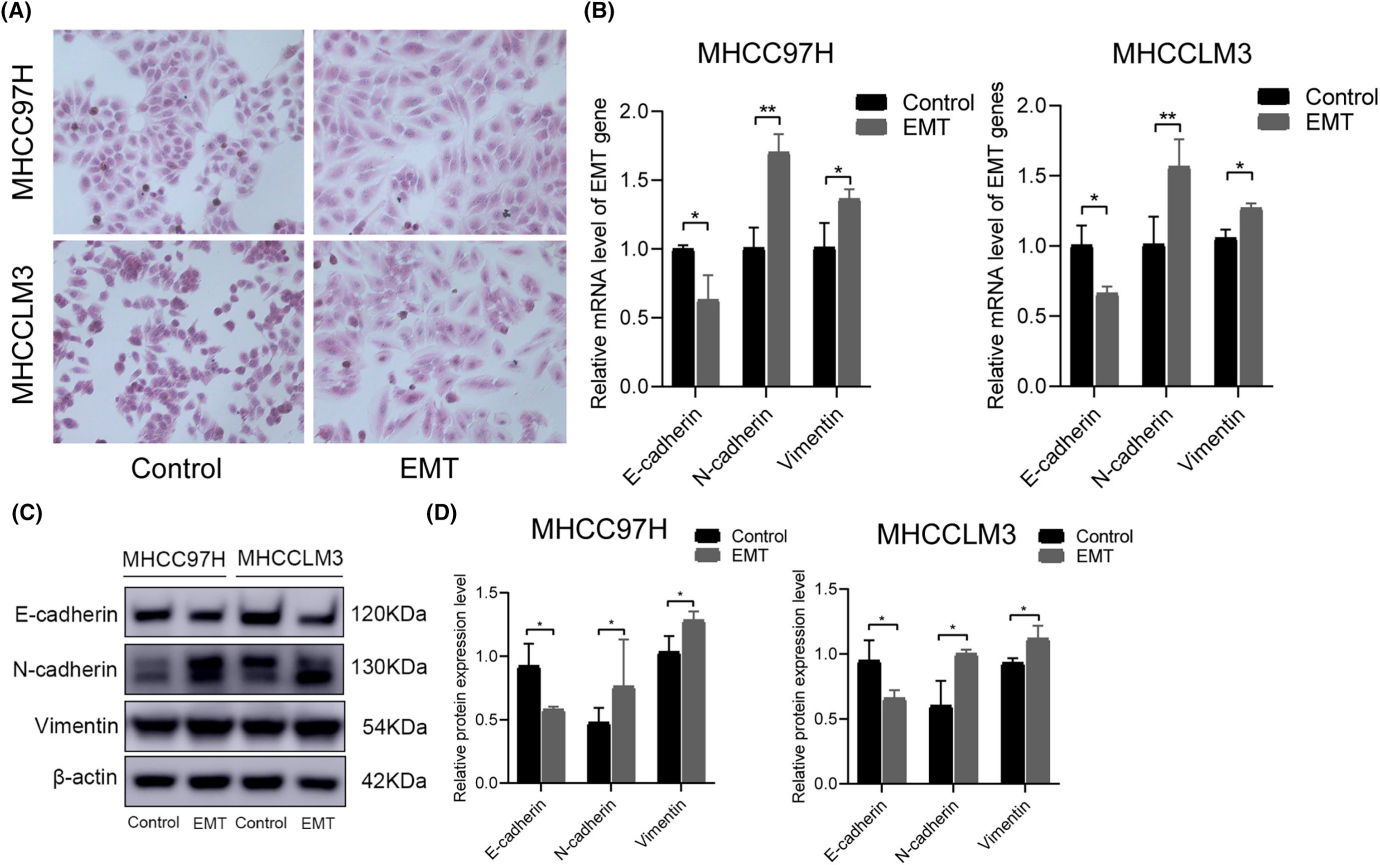
Establishment and verification of HCC EMT model cells. (A) Representative images of H&E staining of HCC EMT model cells. (B) Statistical results on the mRNA expression levels of EMT‐regulated genes. (C, D) Protein electropherogram and statistical results on the protein expression levels of EMT‐regulated genes. All data are presented as mean ± standard deviation (SD; *n* = 3). (*p < 0.05 when compared to the Control group; **p < 0.01 when compared to the Control group).

To observe the morphological changes of the HCC cells during EMT, HE staining was performed. The results showed that the control cells grew well, and displayed an epithelioid form by microscopy, and ovoid, polygonal, or irregular shapes. HCC cells treated with 20 ng/mL TGF‐β1 showed a slender morphology. The number of cells in the EMT model group was significantly increased, and the cells exhibited a long spindle shape (Figure 5A). The expression levels of E‐cadherin, N‐cadherin, and vimentin were detected by RT‐PCR and western blotting assay to verify whether the EMT model was successfully established. Epithelial‐mesenchymal transition (EMT) mainly involved E‐cadherin to N‐cadherin translocation and a rise in vimentin protein expression. The expression levels of E‐cadherin, N‐cadherin, and vimentin in HCC cells treated with TGF‐β1 for 48 h were then examined, as shown in Figure 5B–D. Compared with the Control group, the mRNA and protein expression levels of E‐cadherin were significantly reduced, and those of N‐cadherin and vimentin were significantly increased.

### 3.5 Cytotoxicity assay results

**FIGURE 6:**
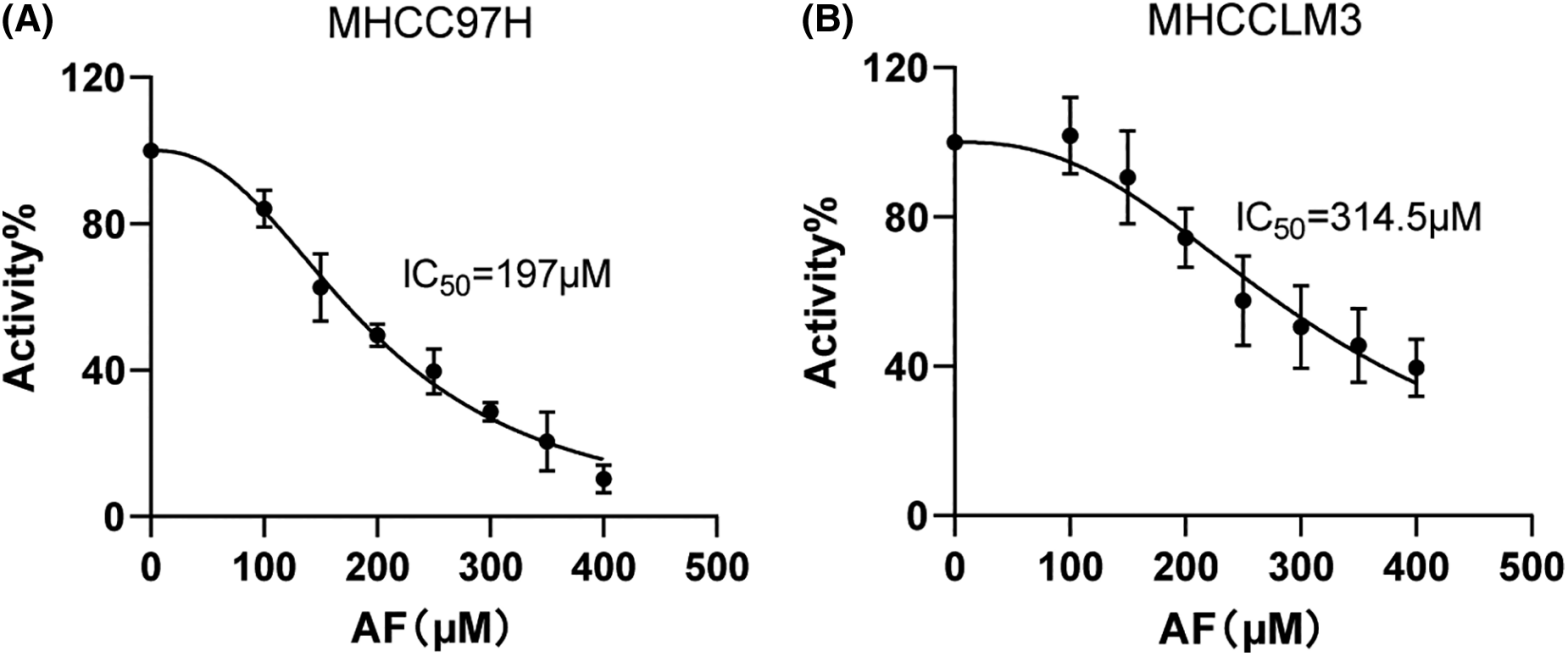
Cytotoxicity of Amentoflavone in HCC cells. (A) Cytotoxicity assay results of Amentoflavone on MHCC97H cell; (B) Cytotoxicity assay results of Amentoflavone on MHCCLM3 cell. All data are presented as mean ± standard deviation (SD; *n* = 4).

Cytotoxicity assay results demonstrated that Amentoflavone inhibited the proliferation of MHCC97H and MHCCLM3 in a dose‐dependent manner (Figure 6). Following treatment with Amentoflavone for 24 h, MHCC97H and MHCCLM3 cells exhibited IC_50_ values of 197 and 314.5 μM, respectively. A lower IC_50_ value signifies that the drug can achieve a 50% inhibition of the target at a lower concentration. Thereafter, all experiments were performed at nontoxic concentrations (below 314.5 μM) of Amentoflavone.

### 3.6 Amentoflavone effectively reversed the TGF‐β1‐induced EMT in HCC cells

**FIGURE 7:**
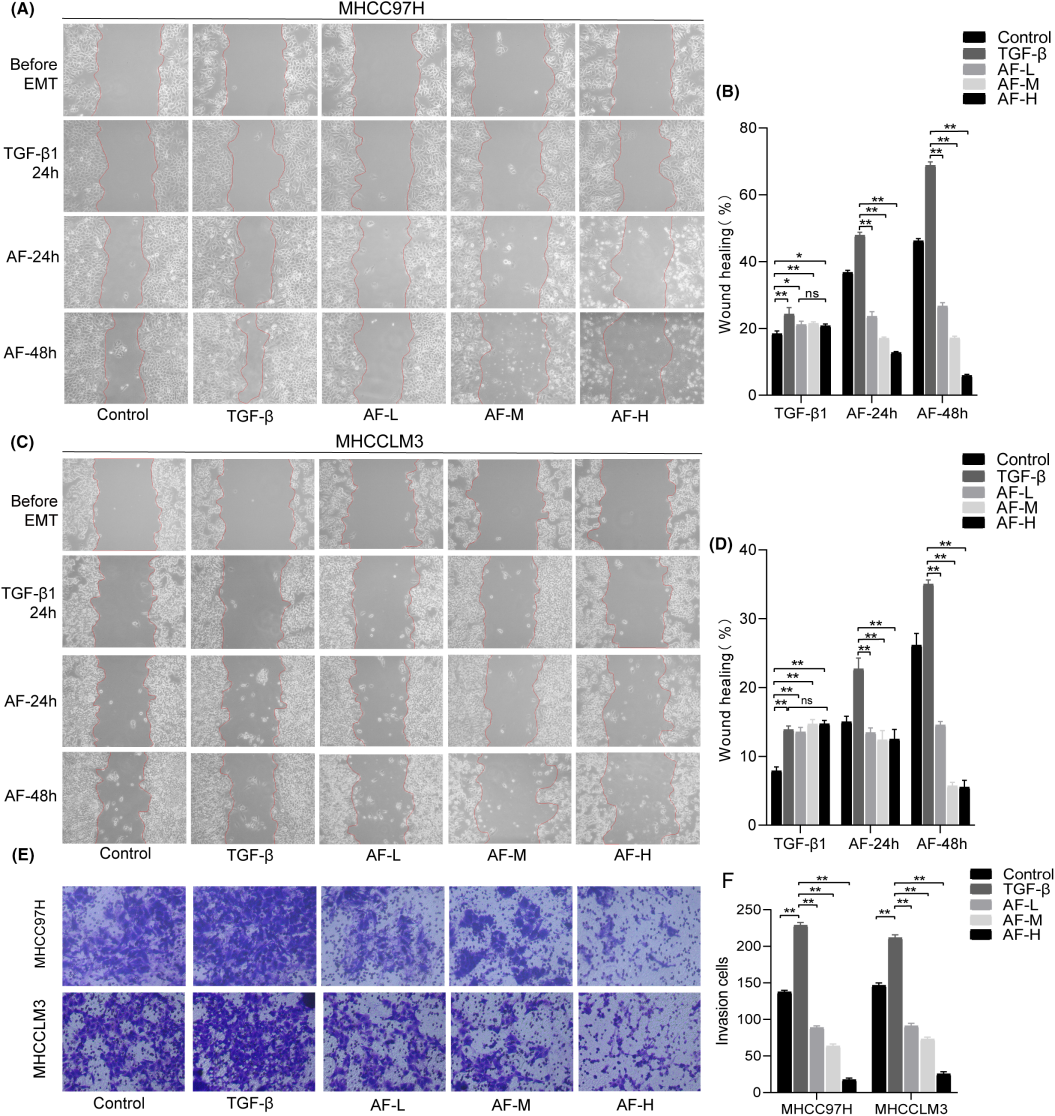
Amentoflavone reverses the EMT in HCC cells. (A–D) Representative images and statistical results of wound healing assay; (E, F) Representative images and statistical results of Transwell assay. All data are presented as mean ± standard deviation (SD; *n* = 3). (*p < 0.05, **p < 0.01, and *n s*., not significant).

The impact of Amentoflavone on the migration and invasion capabilities of HCC cells was assessed through wound healing and Transwell assays. The results of the wound healing assay are illustrated in Figure 7A–D. In comparison to the Control group, the migration rate of HCC EMT model cells was significantly increased after 24 h of TGF‐β1 treatment. After 24 or 48 h of Amentoflavone intervention, the migration rate of HCC cells in the control and EMT model group gradually increased, while the migration rate of cells in the Amentoflavone‐treated group gradually decreased. The effect of Amentoflavone on the migratory capacity of HCC cells was time‐ and dose‐dependent. Notably, after 24 h of 200 or 300 μM Amentoflavone intervention the cells' ability to adhere to the well began to decrease, and after 48 h the cells began to drift away to the well and the migration rate was lower than before. The results of Transwell assay are shown in Figure 7E,F, In comparison to the Control group, the number of cells crossing the matrigel was significantly increased in the EMT model group, whereas the number of cells crossing the matrigel was significantly reduced in the Amentoflavone‐treated group. The effect of Amentoflavone on the invasive capacity of HCC cells acts in a dose‐dependent manner.

### 3.7 Amentoflavone induces apoptosis in HCC cells

**FIGURE 8:**
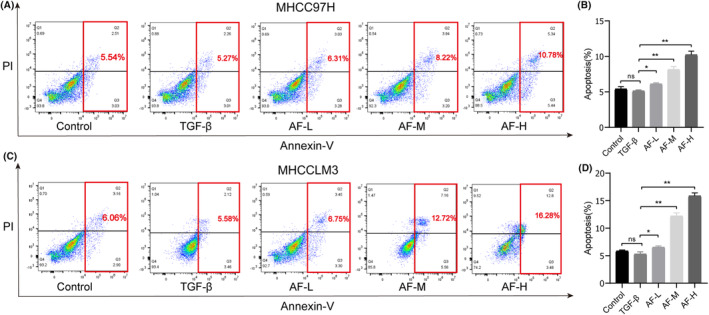
Amentoflavone induces apoptosis in HCC cells. (A) The detection of apoptosis of MHCC97H cells by Annexin V‐FITC/PI staining and flow cytometry; (B) Statistical data chart of apoptosis rate of MHCC97H cells; (C) Detection of apoptosis of MHCCLM3 cells by Annexin V‐FITC/PI staining and flow cytometry; (D) Statistical data chart of apoptosis rate of MHCCLM3 cells. All data are presented as mean ± standard deviation (SD; *n* = 3). (*p < 0.05, **p < 0.01, and *n s*., not significant).

Flow cytometry analysis of annexin V/PI‐stained cells was used to determine whether Amentoflavone induced HCC cell death (Figure 8). In comparison with the control group, the proportion of apoptotic cells were not statistically. In comparison with the EMT group, the proportion of apoptotic cells were significantly higher in the low‐dose Amentoflavone‐treated HCC cells (6.31% vs. 5.27% and 6.75% vs. 5.58% respectively). The proportion of apoptotic cells were significantly higher in the AF‐M and AF‐H group compared with the EMT group. These findings demonstrated that treatment with the Amentoflavone induced apoptosis in the HCC cells.

### 3.8 Amentoflavone‐reversed EMT in HCC cells is mediated by the p53 signalling pathway axis

**FIGURE 9:**
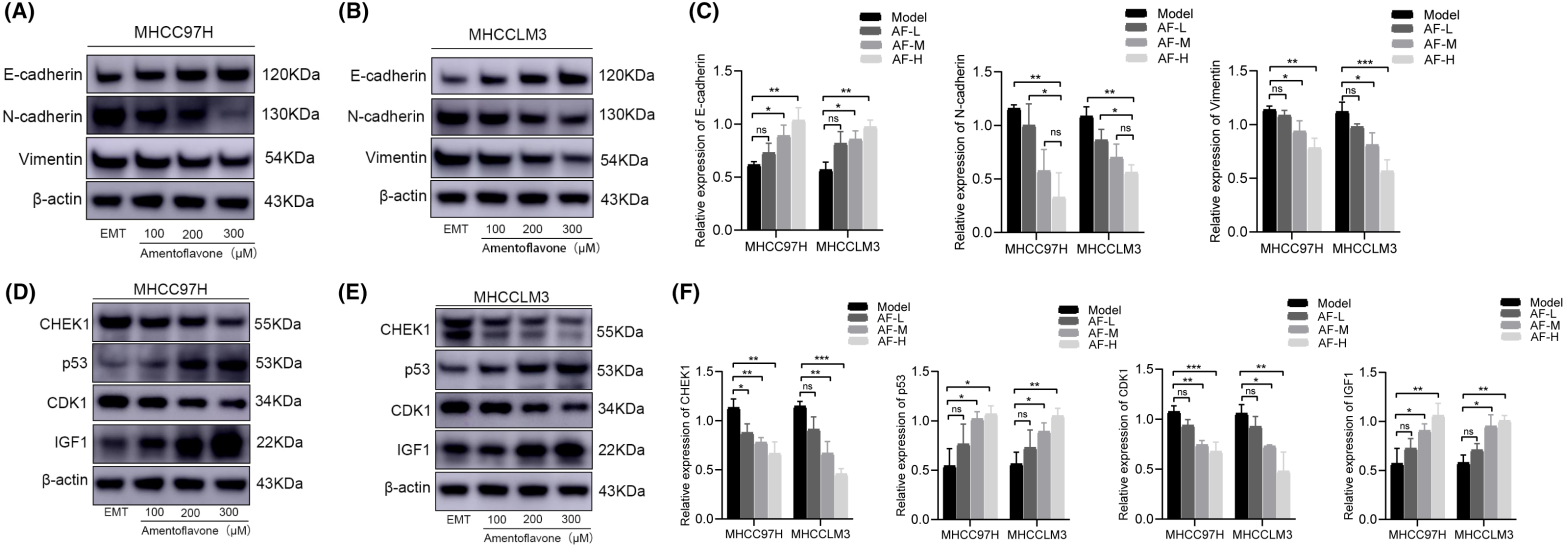
Amentoflavone‐reversed EMT in HCC cells is mediated by the p53 signalling pathway axis. (A, B) Protein electropherogram of EMT‐regulated genes; (C) Statistical results of protein express levels; (D, E) Protein electropherogram of p53 signalling pathway proteins; (F) Statistical results of protein express levels. All data are presented as mean ± standard deviation (SD; *n* = 3). (*p < 0.05 when compared to the Control group; **p < 0.01 when compared to the Control group).

EMT mainly involved E‐cadherin to N‐cadherin translocation and a rise in Vimentin protein expression. Western blotting experiments demonstrated that treatment of Amentoflavone significantly reverses the translocation of E‐cadherin to N‐cadherin and reduces the protein expression of Vimentin. The above results indicate that Amentoflavone can inverse TGF‐β1‐induced EMT in HCC cells. After Amentoflavone intervention, the expression of p53 protein increased with the increase of Amentoflavone dosage. The protein expressions of CHEK1 and CDK1 protein expression levels decreased with an increasing dosage, while IGF1 increased. These results suggest that Amentoflavone‐reversed EMT in HCC cells is mediated by the p53 signalling pathway axis (Figure 9).

## 4 DISCUSSION

EMT is the process by which epithelial cells are converted in a specific way into cells that have a mesenchymal phenotype morphologically and play an important role in tumour metastasis.^21^ EMT is characterized by the loss of cell polarity, decreased expression of epithelial cell markers (such as E‐cadherin), and increased expression of mesenchymal markers (e.g. N‐cadherin and Vimentin). The occurrence of EMT in tumour cells causes them to lose cell polarity and increase cell membrane fluidity, which leads to a decrease in adhesion and an increase in migration capacity.^22^ Tumour cells with increased ability to migrate are more likely to leave their original location and move through the circulatory and immune systems to other parts of the body, where they can relocate and form secondary tumours.

Amentoflavone is initially isolated from the leaves of *Selaginella tamariscina*, *Selaginella rupestris*, and *Ginkgo biloba*.^23^, ^24^ Amentoflavone is also the main ingredient in *Selaginella Doederleinii* Hieron,^25^ which was widely used in Traditional Chinese Medicine to fight cancer. Amentoflavone has a variety of biological activities, such as anti‐inflammatory and neuroprotective effects.^15^, ^26^, ^27^ Amentoflavone exerts anti‐neuroinflammatory effects by inhibiting TLR4/MyD88/NF‐κB and activating Nrf2/HO‐1 pathway in lipopolysaccharide‐induced BV2 microglia.^28^ Li's study demonstrated that Amentoflavone has an antioxidant protective effect.^27^ Amentoflavone is also used in the treatment of ischemic stroke,^29^ Parkinson's disease,^26^ and Alzheimer's disease^30^ because of its neuroprotective effects. Amentoflavone also has therapeutic effects on various types of cancers. In non‐small cell lung cancer cells, Amentoflavone treatment significantly increases the cell population at the G1/G0 phase by decreasing the expression of cyclin D1, CDK4, and CDK6 in both H358 and H1299 cells.^31^ In bladder cancer, AF induces FAS/FASL‐dependent extrinsic apoptosis through increasing pro‐apoptotic protein levels of FAS and FASL.^16^ Amentoflavone exerts antitumor effects by inhibiting colorectal cancer EMT through the miR‐16‐5p/HMGA2/β‐catenin pathway.^32^ These studies provide a lot of evidence that Amentoflavone is a potentially effective multitargeting drug for the prevention and treatment of a variety of cancers. Amentoflavone has a series of molecular targets and the underlying mechanisms are mainly through regulating the expression of different genes involved in cancer cell growth, cell cycle, apoptosis, autophagy, metastasis, angiogenesis, epigenetic modification, etc. However, no studies of Amentoflavone intervention in HCC EMT have been reported. In this study, Amentoflavone, an active ingredient, was innovatively selected to provide some theoretical basis for the treatment of HCC with Amentoflavone.

Previously there have been several reports of EMT‐related genes applied to predict the prognosis of hepatocellular carcinoma.^33^, ^34^, ^35^ However, no studies have been reported to predict the prognosis of hepatocellular carcinoma based on the modulation of EMT‐related genes by active ingredients of drugs. In this study, 14 genes associated with Amentoflavone‐regulated EMT were identified using bioinformatics analysis to predict the prognosis of hepatocellular carcinoma. The TCGA hepatocellular carcinoma cohort was used for internal and external validation and further screening to derive the three core genetic markers, including NR1I2, CDK1, and CHEK1. Patients with hepatocellular carcinoma were categorized into high‐risk and low‐risk groups based on risk scores, and it was found that the prognosis of patients in the high‐risk group was significantly worse than that of the low‐risk group. Furthermore, we found that the three core genetic markers were an independent predictor of 1‐, 3‐, and 5‐year OS in patients with hepatocellular carcinoma. Nomogram and calibration analysis showed that the risk scores for the three genetic markers were in good agreement with the actual observations. These results suggest that a risk score model based on Amentoflavone‐regulated EMT‐related genes has a strong role in predicting the prognosis of hepatocellular carcinoma patients.

The 14 genes screened by bioinformatics were used to construct a protein–protein interaction network, and the protein targets were colour‐coded according to the size of the degree value, with darker protein targets indicating more protein interactions. By KEGG enrichment analysis, it was found that the core genes of Amentoflavone regulation of HCC EMT were mainly enriched in the p53 signalling pathway. p53, the most important tumour suppressor gene, lies at the critical joint of the complex signalling network for stress response, playing a major role in hepatocarcinogenesis.^36^ Further enrichment to three core genes in the p53 signalling pathway, including CHEK1, CDK1 and IGF1. CHEK1 is a DNA damage sensor that regulates cell cycle progression, DNA damage response and DNA replication,^37^, ^38^, ^39^ plays an important role in tumorigenesis, especially in cancer prognosis and tumour phenotype. CHEK1 is also an upstream target protein of the p53 pathway. In our study, bioinformatics analysis revealed that CHEK1 was highly expressed in HCC and was closely associated with tumour biological properties, such as cancer cell invasion and migration, and EMT. Similarly, Roger et al.; reported that a small molecule CHEK1 inhibitor effectively inhibited cancer cell proliferation in human lung and colorectal cancers.^40^ CDK1 (cyclin‐dependent kinase 1) is an important member of the CDK family, which phosphorylates serine/threonine residues of proteins.^41^, ^42^ CDK1 plays a critical role in cancer progression and cancer cell survival and has emerged as one of the potential targets for tumour therapy.^42^, ^43^ It has been reported that CDK1 promotes cancer cell migration and metastasis by phosphorylating proteins such as EZH2 and activating pathways such as Wnt/β‐Catenin and the ERK/GSK3β/SNAI axis.^44^, ^45^ CDK1 also promotes epithelial‐mesenchymal transition and migration of head and neck squamous carcinoma cells by repressing ∆Np63α‐mediated transcriptional regulation.^46^ Insulin‐like growth factor‐1 (IGF‐1) is a peptide growth factor produced mainly by the liver, which can horizontally reflect liver function, and its expression is downregulated in chronic liver disease and hepatocellular carcinoma.^47^ This is consistent with the results obtained from our bioinformatics analysis. IGF‐1 is closely related to cell proliferation, differentiation and apoptosis,^48^ and also plays an important role in the EMT process of tumour cells as well. Inhibiting IGF1/IGF1R pathway can blocks the aggressive phenotype of triple‐negative breast cancer.^49^ Also, IGF‐mediated EMT can delay resistance or re‐sensitize renal cancer to targeted therapies.^50^ In conclusion, both CDK1 and IDF1 are downstream target proteins of the p53 signalling pathway and independently play roles in regulating cellular EMT, proliferation and metastasis. Subsequently, we performed molecular docking prediction of the core genes with Amentoflavone and found that all three core targets docked well with Amentoflavone, suggesting that Amentoflavone has a regulatory effect on these three core genes.

Based on the results of bioinformatics and network pharmacology, we further conducted in vitro experiments to investigate the role of Amentoflavone on the regulation of EMT in HCC cells. We establish a TGF‐β1‐induced full EMT cell model. The success of the model construction was assessed by observing the changes in cell morphology and detecting the mRNA and protein expression levels of EMT‐related genes. Next, the CCK‐8 assay was used to observe the effect of Amentoflavone on the viability of HCC EMT cells, and the results demonstrated a dose‐dependent inhibitory effect of Amentoflavone on the proliferation of HCC EMT cells. The outcomes from both the wound healing assay and Transwell assay revealed that Amentoflavone effectively suppressed the migratory and invasive capabilities of HCC EMT cells. The results of Annexin V‐FITC/PI staining assay showed that the different concentrations of Amentoflavone induces apoptosis in HCC EMT cells. The Western blotting results illustrated that Amentoflavone elicited an up‐regulation in the protein expression levels of E‐cadherin and IGF1, while concurrently down‐regulating the protein expression levels of Vimentin, CHEK1, and CDK1, indicative of its regulatory impact on these key molecular targets. Our experiments showed that Amentoflavone reversed EMT in HCC cells by modulating the p53 signalling axis, which in turn inhibited the invasion and metastasis of hepatocellular carcinoma.

## 5 CONCLUSION

**FIGURE 10:**
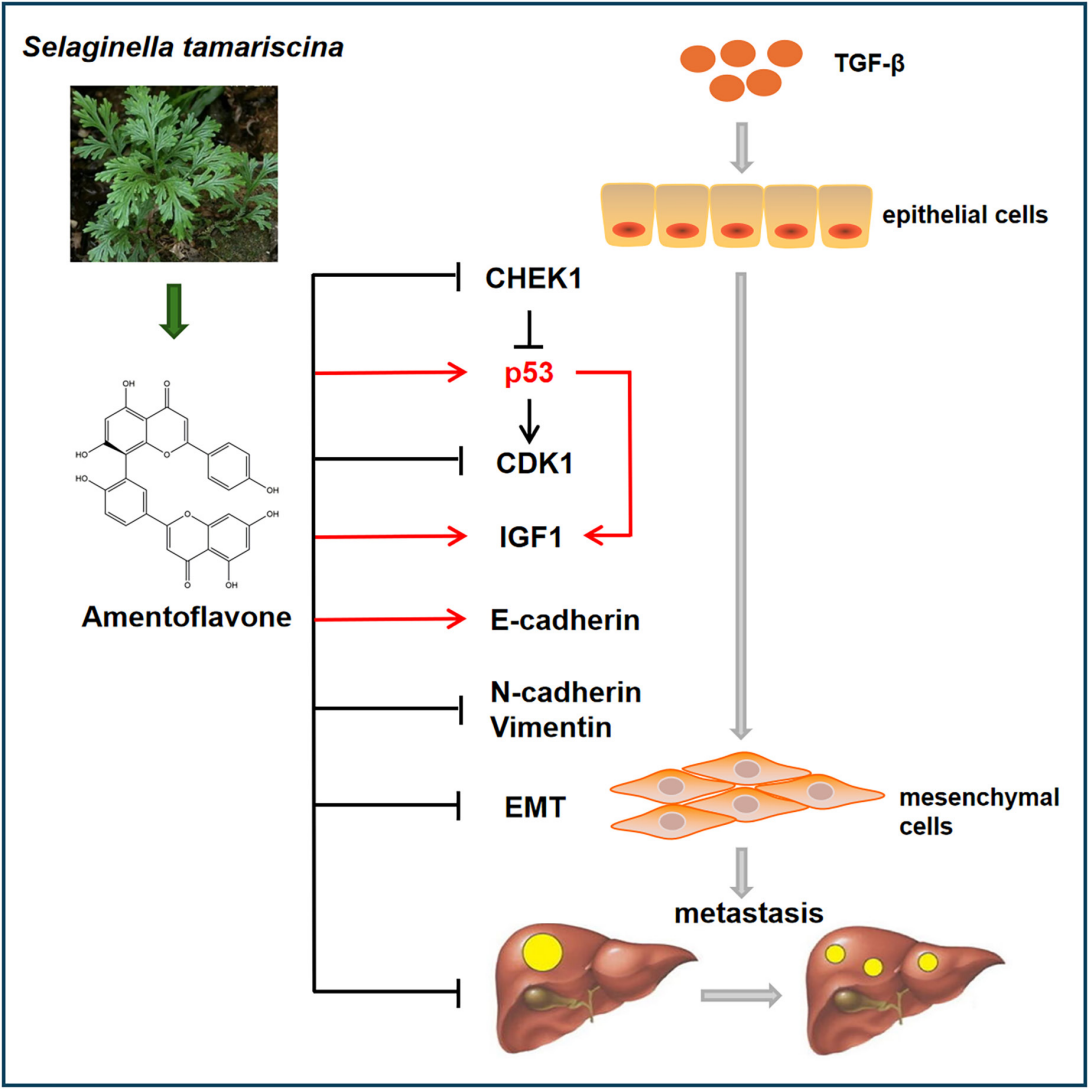
Anti‐epithelial‐mesenchymal transition mechanism of Amentoflavone in TGF‐β1 treated HCC cells.

In summary, in this study, the differential genes of HCC EMT regulated by Amentoflavone were screened by bioinformatics methods, and a prognostic prediction model was constructed that could effectively predict the OS rate of patients with HCC. The signal pathway of Amentoflavone reversing HCC EMT was predicted by network pharmacology and molecular docking technology. Experimental studies further substantiated that the inhibitory effects of Amentoflavone on HCC cell proliferation, migration, and invasion were linked to the p53 signalling pathway axis (Figure 10). Our study provides valuable insights into the role of Amentoflavone in HCC therapy.

## AUTHOR CONTRIBUTIONS

**Hui‐Ying Jian:** Conceptualization (equal); data curation (equal); formal analysis (equal); methodology (equal); project administration (equal); writing – original draft (equal); writing – review and editing (equal). **Jing‐Ting Zhang:** Data curation (equal); methodology (equal); project administration (equal); resources (equal); validation (equal). **Zhuo Liu:** Conceptualization (equal); methodology (supporting); project administration (supporting); resources (supporting); supervision (supporting). **Zhen Zhang:** Conceptualization (equal); formal analysis (equal); methodology (equal); writing – review and editing (supporting). **Pu‐Hua Zeng:** Investigation (lead); project administration (lead); resources (lead); writing – review and editing (lead).

## FUNDING INFORMATION

This study was supported by the National Natural Science Foundation of China (82074425), and the Hunan Provincial Natural Science Foundation (2023JJ40403).

## CONFLICT OF INTEREST STATEMENT

The authors declare that the research was conducted in the absence of any commercial or financial relationships that could be construed as a potential conflict of interest.

## Data Availability

Data available on request from the authors.
